# Supplementary material for: Bistable organic electrochemical transistors: enthalpy vs. entropy
Source: Nat Commun. 2024 Aug 9;15:6819. doi: 10.1038/s41467-024-51001-9 (PMC11316041; doi:10.1038/s41467-024-51001-9)
Supplement: Supplementary file 1 — Supplementary Information [file 41467_2024_51001_MOESM1_ESM.pdf]

Bistable Organic Electrochemical Transistors: Enthalpy vs. Entropy

Lukas M. Bongartz[1*], Richard Kantelberg[1], Tommy Meier[1], Raik Hoffmann[2], Christian Matthus[3], Anton Weissbach[1], Matteo Cucchi[1], Hans Kleemann[1], Karl Leo[1]

[1]IAPP Dresden, Institute for Applied Physics, Technische Universität Dresden, Nöthnitzer Str. 61, 01187 Dresden, Germany

[2]Fraunhofer Institute for Photonic Microsystems IPMS, Center Nanoelectronic Technologies, An der Bartlake 5, 01099 Dresden, Germany

[3]Chair of Circuit Design and Network Theory (CCN), Faculty of Electrical and Computer Engineering, Technische Universität Dresden, Helmholtzstr. 18, 01069 Dresden, Germany

Supplementary Information

Contents

Supplementary Figures **3**

Fig. S1: Bifurcation diagram . . . 3
Fig. S2: Micrographs of solid-state OECTs . . . 4
Fig. S3: Transfer curve with extremely reduced scan rate . . . 4
Fig. S4: Solid-state OECT with Ag/AgCl gate . . . 5
Fig. S5: Bistability in time domain . . . 5
Fig. S6: Transient state retention with gate current . . . 6
Fig. S7: Suppressing the bistability via entropy and enthalpy (log scale) . . . 6
Fig. S8: Long-term validation of suppressed bistability . . . 7
Fig. S9: Transient state retention under enthalpic and entropic dominance . . . 7
Fig. S10: Non-monotonic dependence of the subthreshold swing on temperature . . . 8
Fig. S11: Noise extraction of Schmitt trigger . . . 8
Fig. S12 Ionic circuit of an OECT . . . 8
Fig. S13 Dynamic instability . . . 8
Fig. S14 Chemical potential profile as a cycle process . . . 8
Fig. S15 In-operando thermography study of a solid-state OECT . . . 8
Fig. S16 Subthreshold swing of a bistable OECT (simulation) . . . 8
Fig. S17 Extracted and fitted doping efficiencies α . . . 8
Fig. S18 Effect of the drain voltage . . . 8
Fig. S19 Dynamic response of a bistable system . . . 8
Fig. S20 OECT oscillation . . . 8
Fig. S21 First-order Butterworth filter . . . 8

Supplementary Notes **9**
Supplementary Note 1: Theoretical Framework 9
Supplementary Note 2: Dynamic Instability 11
Supplementary Note 3: Maxwell Construction 14
Supplementary Note 4: Subthreshold Swing 16
Supplementary Note 5: Fitting of Gibbs Free Energy 18
Supplementary Note 6: Doping Efficiency 20
Supplementary Note 7: Effect of the Drain Voltage 22
Supplementary Note 8: Schmitt Trigger 23

Supplementary Figures

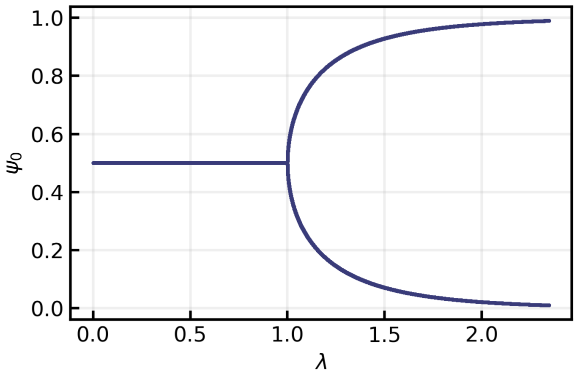

Fig. S1: Bifurcation diagram. For $\lambda > 1$ (Eq. 8), enthalpic contributions to the Gibbs free energy $G(\psi)$ dominate over entropic contributions, which leads to a bifurcation of the equilibrium state at ψ_0. Shown here is the case of $h_{uu} = h_{dd} = 0$ for $\psi = 0.5$.

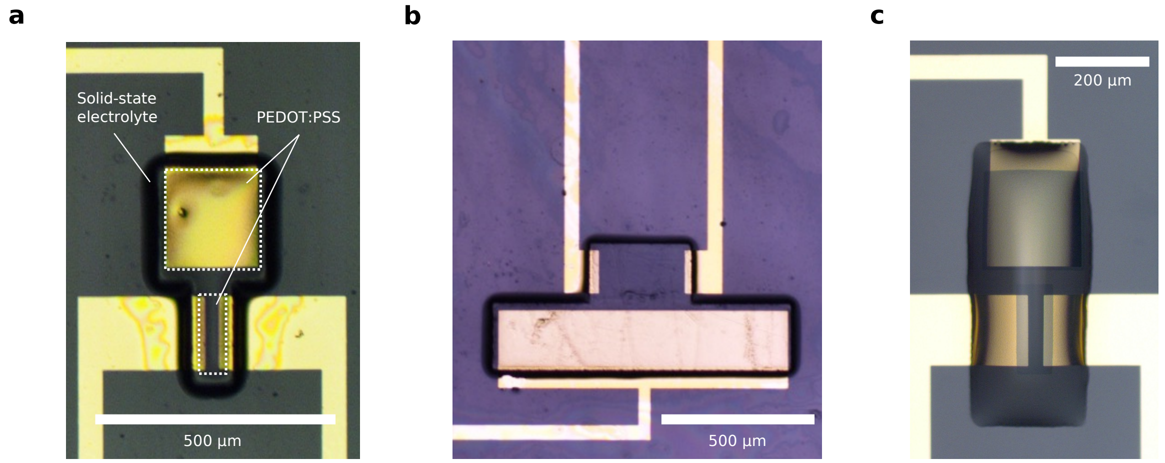

Fig. S2: Micrographs of solid-state OECTs. Lithography-fabricated devices with channel dimensions of (**a**) $L = 30\,\mu\text{m}$, $W = 150\,\mu\text{m}$ and (**b**) $L = 300\,\mu\text{m}$, $W = 150\,\mu\text{m}$. (**c**) Devices fabricated in a hybrid process (lithography and inkjet-printing) with $L = 30\,\mu\text{m}$, $W = 150\,\mu\text{m}$ and modified electrolyte system (hygroscopic KCl additive).

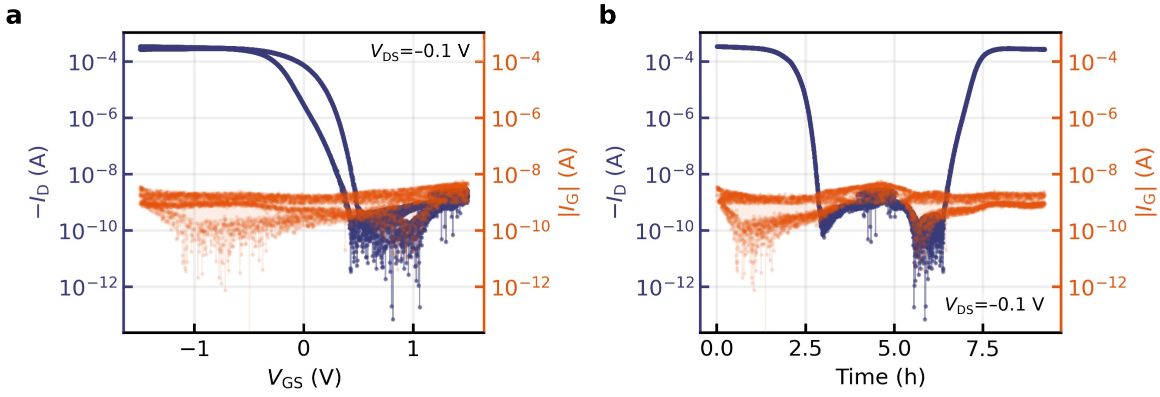

Fig. S3: Transfer curve with extremely reduced scan rate. (a) Transfer curve of the solid-state OECT with a scan rate of $180\,\mu V\,s^{-1}$ with (b) the transient response.

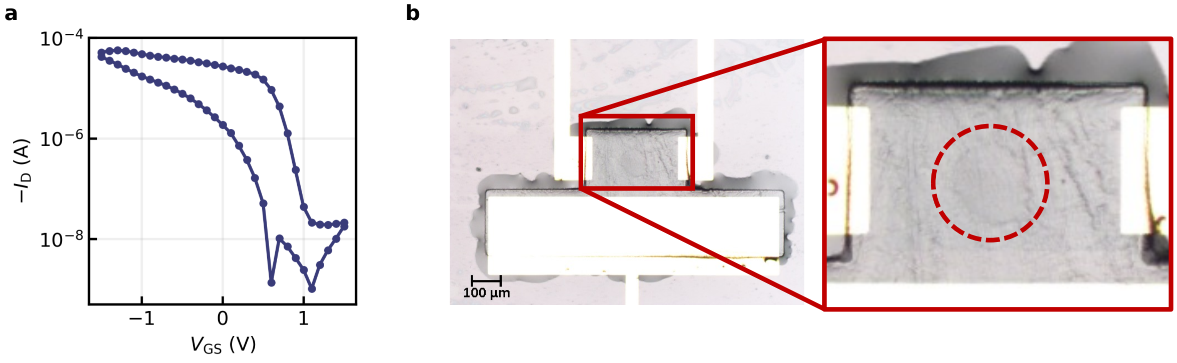

Fig. S4: Solid-state OECT with Ag/AgCl gate. (**a**) Verification of hysteresis when operating with an Ag/AgCl gate. (**b**) Imprint of $80\,\mu m$ diameter gate verifies that the Au side-gate was not in contact.

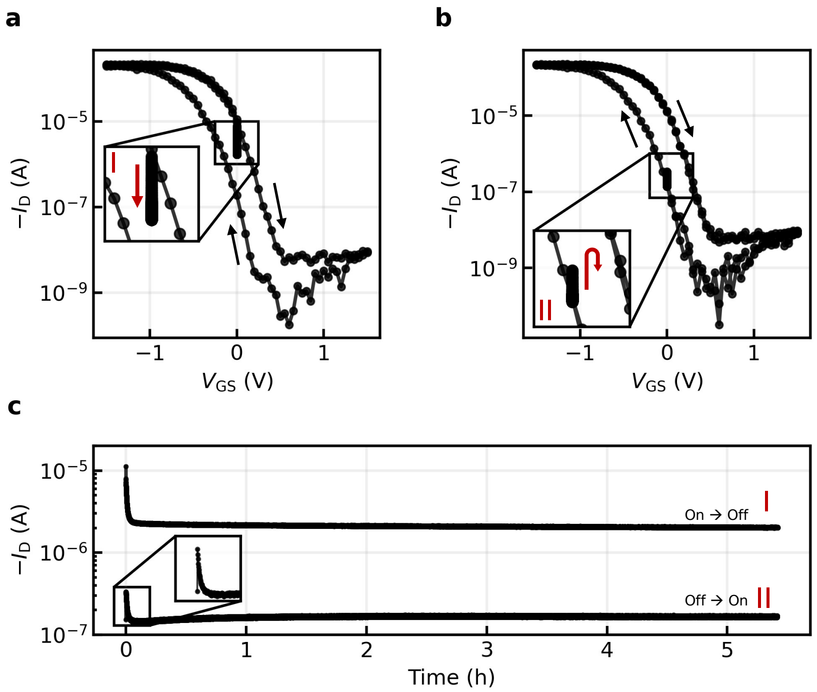

Fig. S5: Bistability in time domain. Operating an OECT and holding a 0 V gate bias approaching from the (**a**) on- or (**b**) off-state reveals (**c**) two coexisting equilibrium states.

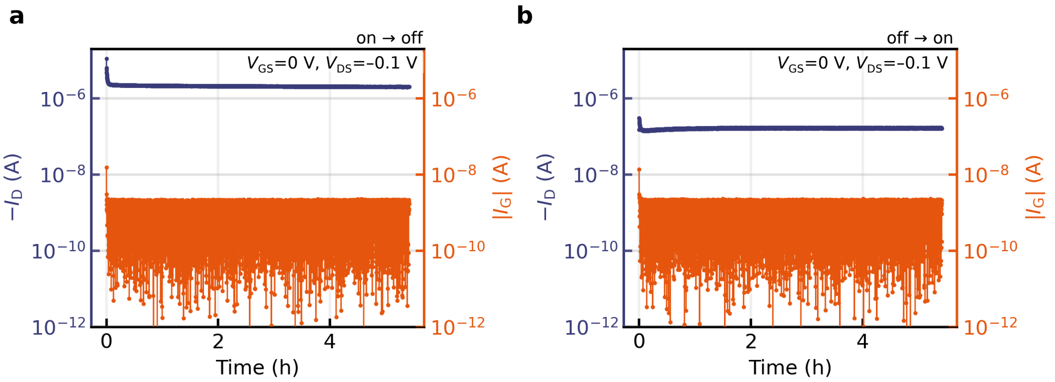

Fig. S6: Transient state retention with gate current. State retention in (a) low-resistance state and (b) high-resistance state including the gate current.

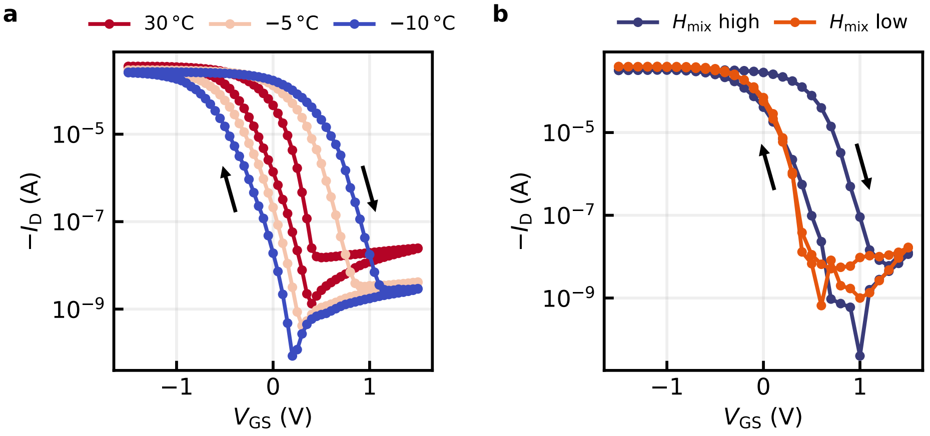

Fig. S7: Suppressing the bistability via entropy and enthalpy (log scale). Data of (**a**) Fig. 3a and (**b**) Fig. 3b in logarithmic scale. Note also the change in subthreshold swing for both cases.

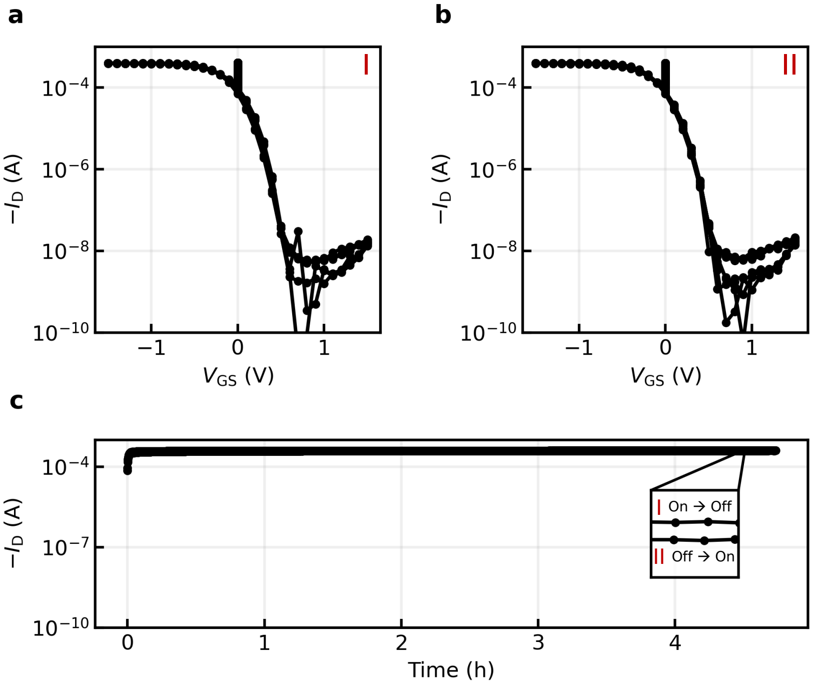

Fig. S8: Long-term validation of suppressed bistability. The experiment of Fig. 1d and S5 was carried out with the system of lowered enthalpy (hygroscopic KCl additive). (**a**) Approaching from the on-state. (**b**) Approaching from the off-state. (**c**) Both tracks approach the same current level, confirming the transition from bi- to monostability.

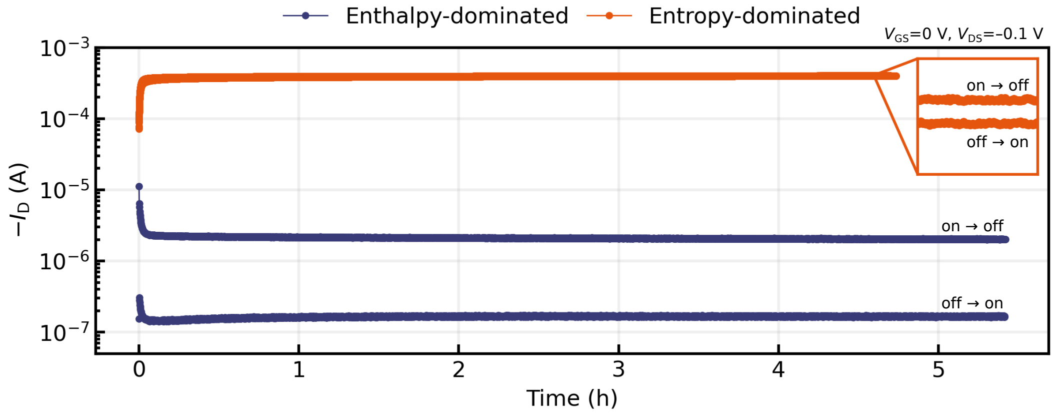

Fig. S9: Transient state retention under enthalpic and entropic dominance. Shielding the enthalpic interactions electrostatically diminishes the bistability, which translates to a suppressed state retention.

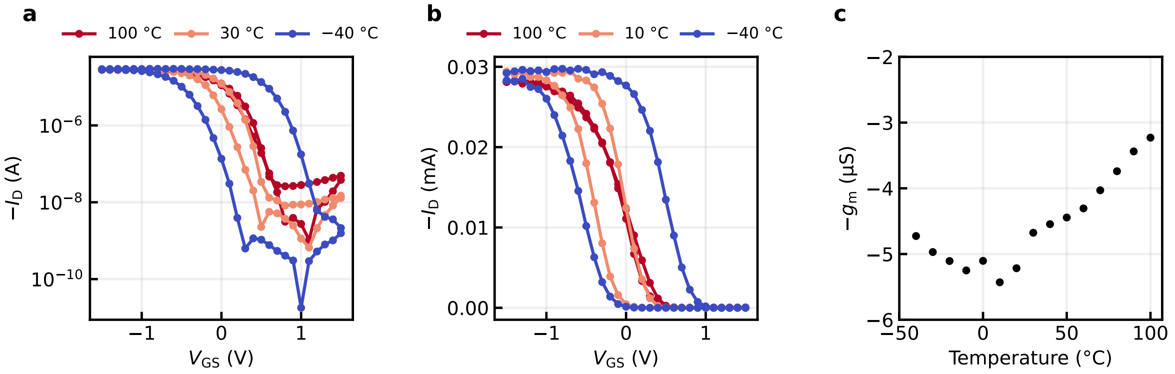

Fig. S10: Non-monotonic dependence of the subthreshold swing on temperature. (**a**, **b**) Temperature-dependent transfer measurements ($V_{DS} = -0.01\,\mathrm{V}$) reveal a non-monotonic progression of the subthreshold swing, going along with a steadily decreasing bistability. (**c**) Similarly, a non-monotonic dependence of the transconductance on temperature is found.

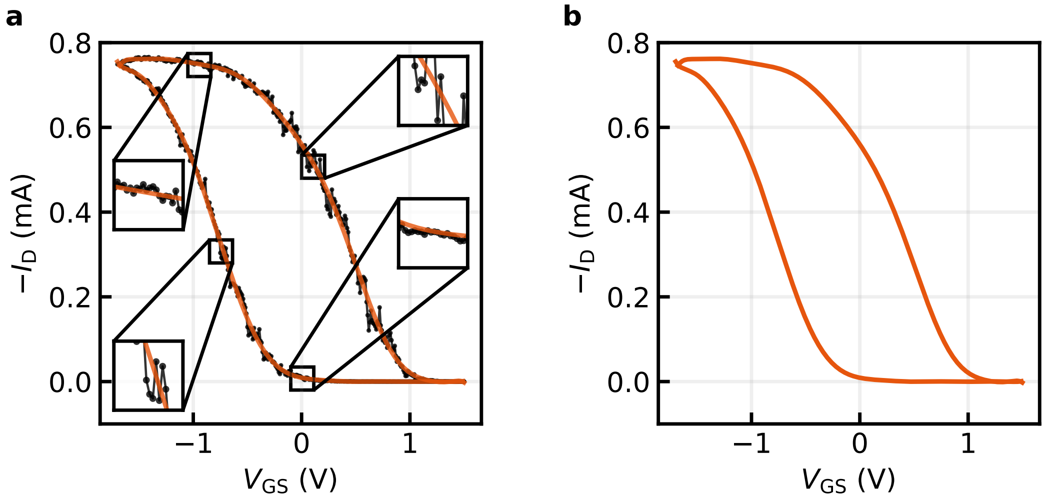

Fig. S11: Noise extraction of Schmitt trigger. (**a**) Analytical function overlaying the output. (**b**) The analytical function reflects the transfer curve with on- and off-state saturation, without overfitting the output noise.

For noise analysis, the output noise is determined against an analytic reference for practical reasons, namely a polynomial fit of order 12. Such high order was necessary to adequately represent the shape of the sweeps, including the saturation of the on- and off-states. The function shows good, balancing overlay with the raw data (Fig. S11a), while at the same time does not over-fit the noise, which would indicate a disproportionately strong noise reduction (Fig. S11b).

Supplementary Notes

Supplementary Note 1: Theoretical Framework

We consider the Gibbs free energy function, defined as

$$G = H - TS, \tag{S1}$$

where H is enthalpy, T is temperature, and S is entropy. Let the channel be composed of doping subunits, where one subunit is the smallest entity that satisfies the doping equation

$$\text{PEDOT:PSS} + \text{C}^{+} + \text{A}^{-} \rightleftharpoons \text{PEDOT}^{+}\text{:PSS} + \text{C}^{+} + \text{A}^{-} + \text{e}^{-}, \tag{S2}$$

where PEDOT:PSS and PEDOT^{+}:PSS are the initial and doped state of the OMIEC, and C^{+} and A^{-} are the electrolyte cat- and anions. Given this, we can regard Eq. S1 as a function of the relative share of doped units ψ:

$$\psi = \frac{N_{\text{doped}}}{N_{\text{doped}} + N_{\text{undoped}}} = \frac{N_{\text{doped}}}{N_{\text{tot}}}, \tag{S3}$$

where N_{doped} and N_{undoped} are the number of doped and undoped sites and N_{tot} is the total number available. For the binary system, the Gibbs free energy function follows as

$$G(\psi) = \frac{\tilde{G}(\psi)}{N_{\text{tot}}} = H^{0}(\psi) + H_{\text{tr}}(\psi) - TS_{\text{tr}}(\psi), \tag{S4}$$

with the enthalpic and entropic terms as

$$H^{0}(\psi) = \psi\mu_{\text{d}}^{0} + (1-\psi)\mu_{\text{u}}^{0}, \tag{S5}$$

$$H_{\text{tr}}(\psi) = \frac{Z}{2}\left(w_{dd}\psi^{2} + w_{uu}(1-\psi)^{2} + 2w_{du}\psi(1-\psi)\right), \quad \text{and} \tag{S6}$$

$$S_{\text{tr}}(\psi) = -k_{\text{B}}(\psi\ln(\psi) + (1-\psi)\ln(1-\psi)). \tag{S7}$$

$H^{0}(\psi)$ denotes the standard enthalpy, while $H_{\text{tr}}(\psi)$ and $S_{\text{tr}}(\psi)$ are the enthalpy and entropy associated with the state transitions underlying Eq. S2. μ_{d}^{0} and μ_{u}^{0} are the standard chemical potentials of doped and undoped sites, Z is the coordination number, and w_{dd}, w_{uu}, and w_{du} express the interaction strength between doped sites, undoped sites, and between the two. k_{B} is the Boltzmann constant. The chemical potential follows as

$$\begin{aligned}\mu(\psi) &= \left(\frac{\partial G(\psi)}{\partial \psi}\right)_{p,T} \\ &= \mu_{\text{d}}^{0} - \mu_{\text{u}}^{0} + Z\left(w_{dd}\psi + w_{uu}(\psi-1) - w_{du}(2\psi-1)\right) + k_{\text{B}}T\ln\left(\frac{\psi}{1-\psi}\right)\end{aligned} \tag{S8}$$

and relates to the gate-source voltage V_{GS} via the electrochemical potential as defined in Eq. 7. Since the doping parameter ψ translates to the drain current I_{D}, the transfer curve of an OECT can be seen as a direct consequence of the underlying Gibbs free energy function. For an entropy-dominated system, $G(\psi)$ is a parabola-shaped potential with a single minimum, i.e., a single thermodynamic equilibrium state (Fig. 1a). However, for a system of dominating enthalpy, the single equilibrium state bifurcates, given rise to a partially negative curvature in

$G(\psi)$:

$$\left(\frac{\partial^2 G(\psi)}{\partial \psi^2}\right)_{p,T} \leq 0 \tag{S9}$$

$$Z(w_{dd} + w_{uu} - 2w_{du}) \leq \frac{k_\mathrm{B}T}{\psi(\psi-1)} \tag{S10}$$

$$\lambda = \frac{Z(w_{dd} + w_{uu} - 2w_{du})}{k_\mathrm{B}T} \cdot \psi(\psi-1) \geq 1 \quad \text{with} \quad \psi \in [\psi_i, 1-\psi_i], \tag{S11}$$

where ψ_i and $1-\psi_i$ are the inflection points of $G(\psi)$. Doping concentrations in this ψ-range are unstable and decompose into two coexisting equilibrium states with positive curvature in $G(\psi)$. In this situation, the chemical potential is non-monotonic, having a range of inverted slope between its local extrema at ψ_i and $1-\psi_i$ that cannot contribute to the static transfer curve of an OECT. The quantity λ can in this sense be interpreted to express the degree of bistability present in the system, as it sets enthalpic and entropic contributions in relation to one another. We provide an interactive simulation tool under Ref. 1 to illustrate these relationships.

Given that a single doping unit will involve multiple individual components (e.g., PEDOT units, PSS units, ions), we approximate the standard chemical potentials with $\mu_\mathrm{d}^0 \approx \mu_\mathrm{u}^0$, from which an equilibrium at $\psi = 0.5$ follows for the ideal gas scenario. Apart from that, it is worth noting that the approach we take here is based on the assumption of a lattice structure, where Z is the according coordination number. Obviously, the OMIEC's microstructure does not correspond to a period lattice but is instead of a much more disordered nature, which translates to the interlacing of doping units. Accordingly, Z appears as an unknown quantity that we cannot explicitly separate from the interaction parameters, but only consider the product with

$$h_i = Z \cdot w_i \quad \forall \quad i \in \{dd, uu, du\}, \tag{S12}$$

where for Z we expect values of typical scale for three-dimensional systems, i.e., 2 to ~ 8.

Supplementary Note 2: Dynamic Instability

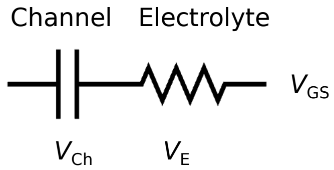

Fig. S12: Ionic circuit of an OECT.

With ψ describing the relative proportion of doped units in the channel, the associated charge follows as

$$\varphi = \psi \cdot e \cdot N_{\mathrm{tot}}. \tag{S13}$$

As the chemical potential $\mu(\varphi)$ is related to V_{GS} through Eq. 7, the inverse derivative of $\mu(\varphi)$ has the character of a capacitance,

$$\frac{\partial \varphi}{\partial \mu(\varphi)} = C(\varphi). \tag{S14}$$

From this, we can express the time dependence of φ through

$$\frac{\partial \varphi}{\partial t} = C(\varphi)\frac{\partial \mu(\varphi)}{\partial t}. \tag{S15}$$

To now understand the dynamic behavior of the channel during a transfer scan, we take the approach of Bernards et al.[2] and describe the system as a series connection of capacitor and resistor, where we assume a non-polarizable gate electrode (e.g., Ag/AgCl) for the sake of simplicity (Fig. S12). Given this system, $\frac{\partial \varphi}{\partial t}$ (Eq. S15) corresponds to the gate current I_{G}, which itself is determined by the effective voltage through the electrolyte V_{E}:

$$I_{\mathrm{G}} \leftrightarrow \frac{\partial \varphi}{\partial t} \tag{S16}$$

$$= \frac{1}{R_{\mathrm{E}}} V_{\mathrm{E}} \tag{S17}$$

$$= \frac{1}{R_{\mathrm{E}}}(V_{\mathrm{GS}} - V_{\mathrm{Ch}}). \tag{S18}$$

Obviously, the dynamic behavior of I_{G} during a V_{GS}-sweep also determines the charge in the channel and therefore, I_{D}. When further considering that the channel potential is predominantly determined by the chemical potential $\mu(\varphi)$, the OECT dynamics can be described through the first-order differential equation

$$I_{\mathrm{D}} \leftrightarrow \frac{\partial \varphi}{\partial t} = \frac{1}{R_{\mathrm{E}}}(V_{\mathrm{GS}} - \mu(\varphi)). \tag{S19}$$

For a given bistability (Eq. 8), one can approximate the bistable potential function as a polynomial of order 4, namely

$$G(\psi) \leftrightarrow V(x) = \alpha x^4 - \beta x^2, \quad \text{where} \quad \alpha, \beta > 0. \tag{S20}$$

Given such a potential (Fig. S13a), one can understand the dynamic behavior of the bistable

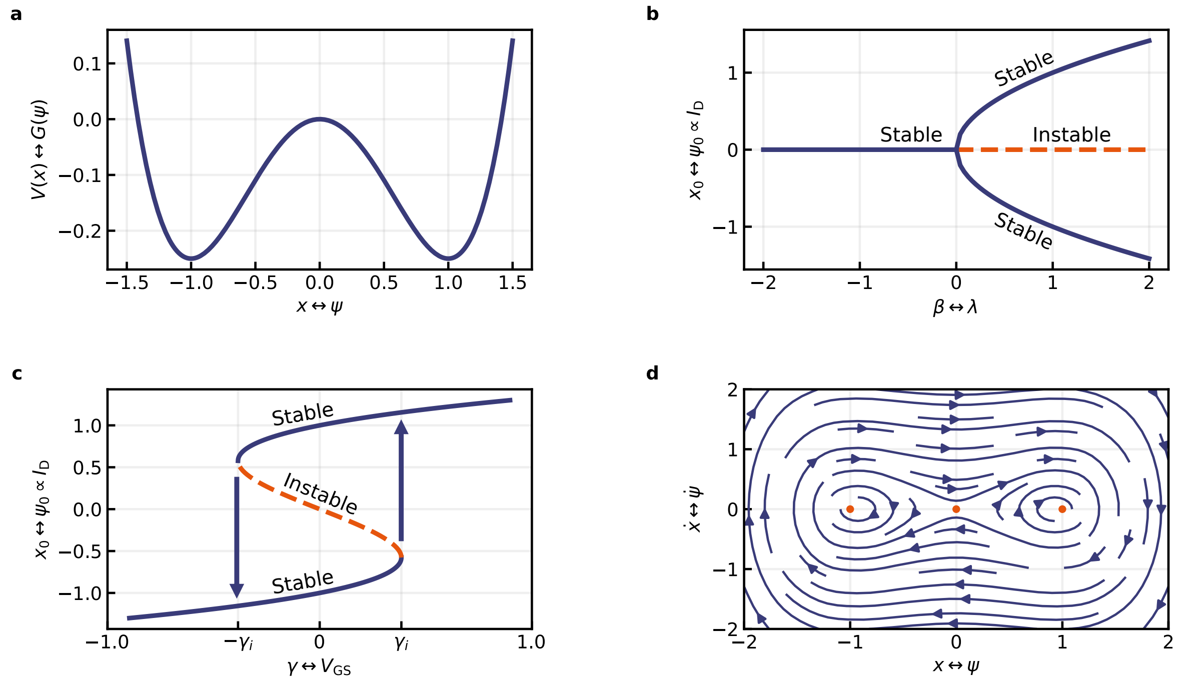

Fig. S13: Dynamic instability. (**a**) $G(\psi)$ is approached by a fourth-order polynomial $V(x)$, according to Eq. S20 ($\alpha = 0.25, \beta = 0.5$). (**b**) Increasing β effects a pitchfork bifurcation of the equilibrium state for $\beta > 0$. (**c**) For an external force γ, stable and instable equilibrium regimes occur, yielding a hysteresis. (**d**) Phase portrait visualizing the dynamics of the bistable system, showing an unstable fixed point in between two stable fixed points.

system by comparing it to a damped, non-linear oscillator. With including a damping proportional to the velocity, it follows from Newton's second law that

$$\ddot{x} = -\frac{\partial V(x)}{\partial x} - \eta\dot{x}, \quad \text{which for strong damping leads to} \tag{S21}$$

$$\dot{x} = -\frac{\partial V(x)}{\partial x} = -4\alpha x^3 + 2\beta x \tag{S22}$$

on the new time axis $t \rightarrow t\eta^{-1}$. Eq. S22 is equal to the force driving the system into its equilibrium states, i.e., the chemical potential $\mu(\psi)$, and as such, is a gradient system. This implies asymptotically stable fixed points of $V(x)$ at $x_0 = \pm\sqrt{\beta(2\alpha)^{-1}}$ and an instable point at $x_0 = 0$, leading to bifurcation depending on β (Fig. S13b). With the two stable points being separated by an instable point, bistable operation follows. When the system is deflected from equilibrium by a constant, external force γ, Eq. S22 turns to

$$\dot{x} = -\frac{\partial V(x)}{\partial x} = -4\alpha x^3 + 2\beta x + \gamma, \tag{S23}$$

leading to bistability in the interval of $\gamma \in (-\gamma_i, \gamma_i)$ with $\gamma_i^2 = 8\beta^3(27\alpha)^{-1}$ (Fig. S13c). The parameter γ will push the system always on a stable path, until either γ_i (from the bottom) or $-\gamma_i$ (from the top) is reached, upon which the transition follows on a very short time scale. This dynamic behavior is visualised by the phase portrait in Fig. S13d, showing the two stable fixed points sideways to the unstable fixed point. We further expand on this reasoning in Supplementary Note 7 to study the dynamic response of the system to a periodic bias.

Transferring this understanding to the OECT system is now straightforward. The double-

well potential $V(x)$ approximates the Gibbs free energy function $G(\psi)$, having its equilibrium state at ψ_0 for the monostable (entropy-dominated) system and at ψ_0 and $1-\psi_0$ for the bistable (enthalpy-dominated) case. The bifurcation parameter β relates to the balance between these forces, entropy and enthalpy, and thus to λ of Eq. 8, while the external force γ corresponds to the gate voltage V_{GS}. It follows that any system obeying the first-order ordinary differential equation derived above will show bistable operation, if the underlying potential function features the topology of Eq. S20 (double-well potential). It further follows from this argumentation that a depletion mode device must necessarily show non-volatile hysteresis in the loop direction of Fig. 1c. That is, a low-resistance state followed by a high-resistance state, when switching from on to off.

Supplementary Note 3: Maxwell Construction

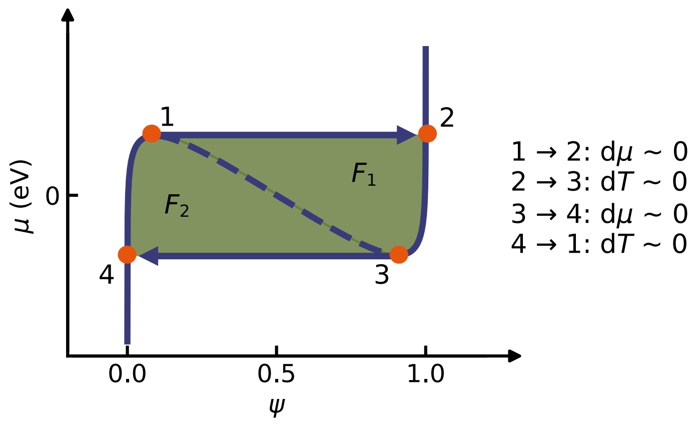

Fig. S14: Chemical potential profile as a cycle process.

We show that the profile of $\mu(\psi)$ can be considered as a cycle process[3]. As laid out in the main text and in Supplementary Note 1, we assume similar standard chemical potential for doped and undoped units, which allows us to approximate the idealized chemical potential point-symmetric around $(\psi, \mu(\psi)) = (0.5, 0\,\text{eV})$. The first law of thermodynamics dictates that

$$\mathrm{d}U = \delta Q + \delta W, \tag{S24}$$

with the internal energy U and δQ and δW the heat exchanged and work performed. The second law of thermodynamics demands for a reversible process that

$$\delta Q = T\mathrm{d}S, \tag{S25}$$

while the performed chemical work corresponds to

$$\delta W = \int \mu(\psi)\mathrm{d}\psi, \quad \text{yielding} \tag{S26}$$

$$\mathrm{d}U = T\mathrm{d}S + \int \mu(\psi)\mathrm{d}\psi \quad \text{and} \tag{S27}$$

$$\mathrm{d}(U - TS) = -S\mathrm{d}T + \mu(\psi)\mathrm{d}\psi. \tag{S28}$$

With the Helmholtz free energy defined as

$$F = U - TS, \quad \text{it follows that} \tag{S29}$$

$$\mathrm{d}F = -S\mathrm{d}T + \mu(\psi)\mathrm{d}\psi, \tag{S30}$$

where the first term expresses the exchanged heat and the second the performance of chemical work, corresponding to F_1 and F_2 in Fig. S14:

$$F_1 = F_2 \tag{S31}$$

$$\int_1^2 \mu_{1\to2}(\psi)\ \mathrm{d}\psi = \int_3^4 \mu_{3\to4}(\psi)\ \mathrm{d}\psi. \tag{S32}$$

This reasoning is similar to the concept of Maxwell constructions in the context of phase transitions of non-ideal gases, where the work done during expansion and compression must be equal. Here, however, we refer to the chemical work. From Eq. S30 follows that the

performance of such work must necessarily be associated with the consumption/release of heat, which we validate experimentally in Fig. 3g and S15.

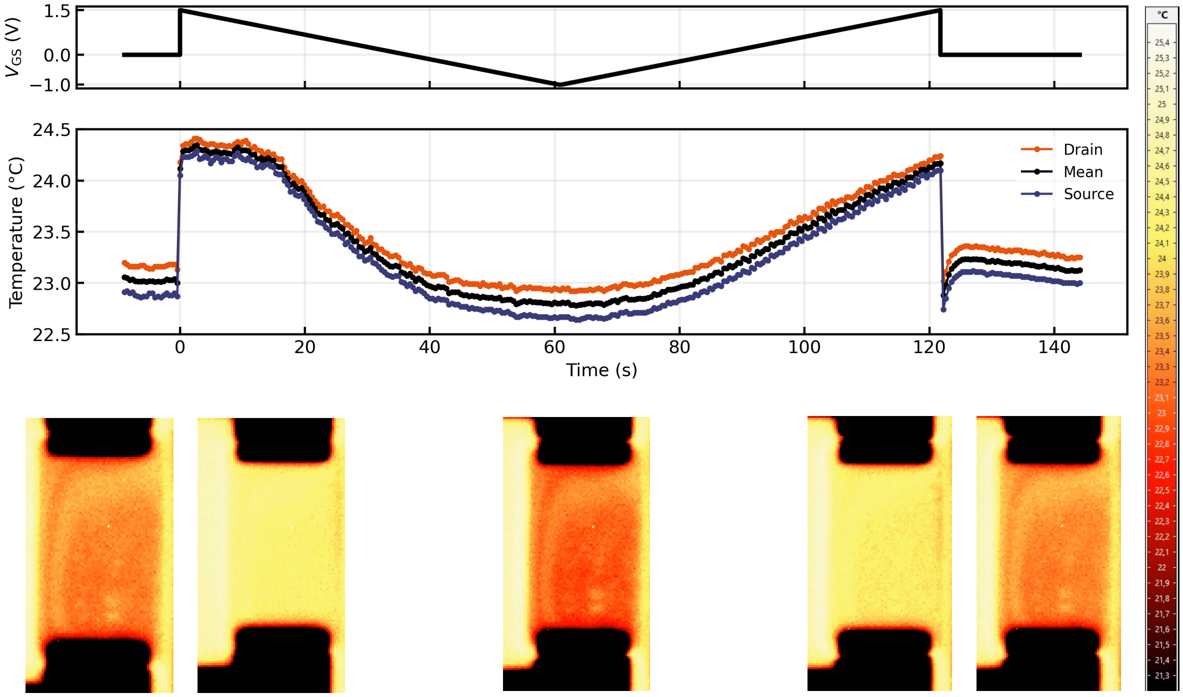

Fig. S15: In-operando thermography study of a solid-state OECT. Heat consumption is observed when switching the OECT from off to on ($V_{GS} = 1.5$ to -1.0 V, $V_{DS} = -0.1$ V). For the time being, differences between source and drain electrodes are omitted and the temperature is averaged over the channel. We attribute the temperature difference, which in the on-state is ~ 0.3 K, to a shift of the doping process towards the source electrode, at which charge injection takes place.

Supplementary Note 4: Subthreshold Swing

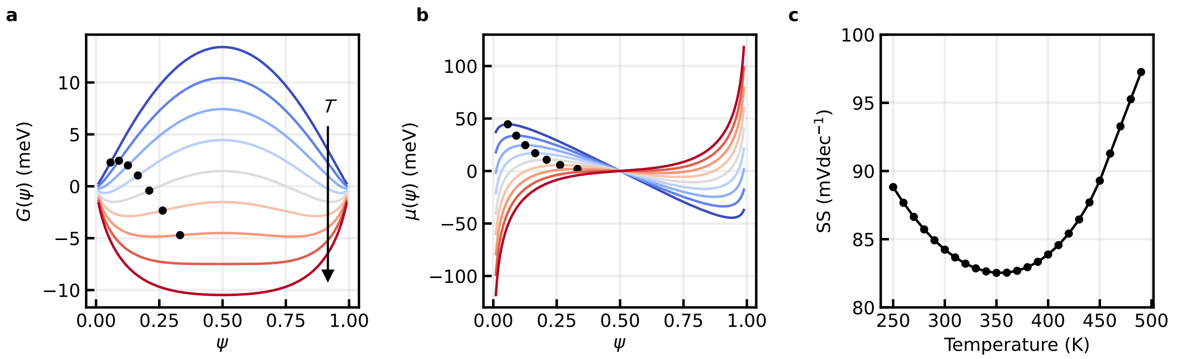

Fig. S16: Subthreshold swing of a bistable OECT (simulation). With a bistable Gibbs free energy function (**a**), the chemical potential (**b**) is non-monotonic. During the transition between the on- and off-state, the total electrochemical potential is constant, implying that chemical potential and electrostatic potential balance (Eq. S33). This effect decreases with rising temperature, causing a non-monotonic progression of the subthreshold swing with temperature. We express this relationship through Eq. S34, which is modelled in (**c**).

As laid out before, for a present bistability, we can assume a Maxwell construction for $\mu(\psi)$ in the regime $\psi \in [\psi_i, 1-\psi_i]$, where the total electrochemical potential is constant during each transition (Fig. S14). Following Eq. 7, this is the result from a balance between the chemical potential resulting from $G(\psi)$ and the electrostatic potential from the gate voltage V_{GS}:

$$\bar{\mu} = \frac{\partial G(\psi)}{\partial \psi} + fe(V_{\mathrm{GS}} - V_{\mathrm{Ch}}) = \text{const.} \tag{S33}$$

This understanding can be transferred to the subthreshold swing (SS), which defines the minimum gate voltage required to change the drain current by one order of magnitude in the subthreshold region. In the ideal case, the subthreshold swing is a purely diffusion-controlled quantity, being inversely proportional to the thermal voltage $\frac{k_{\mathrm{B}}T}{e}$, which defines the typically expected increase with temperature[4]: As the temperature increases, a progressively larger gate potential is required to change the drain current by one order of magnitude, since diffusion deprives charge carriers from their effective modulation. For the bistable OECT system, however, another effect is to be considered: With the non-monotonic chemical potential compensating the electrostatic gate potential, the subthreshold swing is subject to a second influence, which ought to be stronger at lower temperatures. Thus, the subthreshold swing is expected to obey

$$\mathrm{SS}(T) \approx \frac{\ln(10)}{e}\left(\left(\left.\frac{\partial G(\psi,T)}{\partial \psi}\right|_{\psi=\psi_i}\right)_{p,T} + k_{\mathrm{B}}T\right), \tag{S34}$$

where we can assume a doping efficiency of $f \approx 1$, motivated by the low state occupancy in the subthreshold region. The chemical potential is evaluated at its local maximum ψ_i, which is due to the fact that the subthreshold swing for the PEDOT:PSS-based depletion mode OECT is considered for its transition from on to off (Fig. S16a,b). ψ_i in this case is the first thermodynamically stable point, where at the same time, chemical and electrostatic potential are just balanced. Eq. S34 is modelled in Fig. S16c, showing the non-monotonic dependence

on temperature: As long as enthalpy is dominant and bistability is present, the subthreshold swing decreases with rising temperature. From the point on where enthalpic and entropic contributions are balanced, however, the subthreshold swing increases with $k_\mathrm{B}T$.

Supplementary Note 5: Fitting of Gibbs Free Energy

Calculating and fitting the experimental Gibbs free energy data was carried out on the basis of the procedures of Ref. 5. However, certain refinements were necessary to account for the bidirectional device operation and the resulting bistability underlying this work. In particular, we model the Gibbs free energy profiles of the data sets of Fig. 3a (entropy-controlled) and b (enthalpy-controlled). $G(\psi)$ can be extracted from the transfer curve measurements via integration. Especially for the low-temperature regime, however, the given electrochemical window does not allow to capture both the on- and the off-state symmetrically, which may hinder appropriate data integration. To account for this, we perform an analytic continuation of the data in the on-state, by fitting the two branches with a generalized logistic function of the form

$$f(x) = \frac{I_{\text{on}}}{(1 + e^{-b\cdot(x-c)})^v} + I_{\text{off}}, \tag{S35}$$

where x refers to V_{GS} and the fitting parameters b, c, and v account for the fitting functions slope, midpoint, and sharpness. The normalized data sets are then extended in an equidistant manner to achieve symmetry around the midpoints at $\psi = 0.5$. An example is shown in Fig. 3c, where the dark blue data set is continued with the light blue data points. Obviously, this approach preempts any processes potentially occurring at more negative gate voltages that are not covered by the experimental data, like the formation of bipolarons. However, since such processes are not described by our model anyway at this point, the analytic continuation of the on-state as carried out here represents a valid approximation. Given this, the reasoning of Supplementary Note 3 is applied by shifting the data sets to have partial integrals of equal size around $\mu(\psi) = 0\,\text{eV}$ (Fig. 3c). Note that this approach is underpinned by the approximately equal heat exchange for the two sweeps in the thermography study (Fig. 3g and S15). Data integration then yields the experimental Gibbs free energy profiles shown in Fig. 3d and e with hollow points.

For fitting, the model equations (Eq. 4-6) were subjected to polynomial decomposition to then fit the equation

$$g(\psi) = \frac{1}{\alpha}\left(k_{\text{B}}T(\psi\ln(\psi) + (1-\psi)\ln(1-\psi)) + p\psi^2 + q\psi + r\right) \tag{S36}$$

with a weighted Levenberg-Marquardt algorithm, where α accounts for the fudge factor of Eq. 7. Data points were weighted according to

$$w_i = \left(\frac{d\mu_i}{d\psi_i}\right)^2, \tag{S37}$$

before being normalized. From Eq. S36, the h-parameters can be extracted by considering that the Gibbs free energy function can be fully described by three points

$$g_0 = G\left(\psi = 0\right) = \mu_u^0 + \frac{1}{2}h_{uu}, \tag{S38}$$

$$g_1 = G\left(\psi = 1\right) = \mu_d^0 + \frac{1}{2}h_{dd}, \quad \text{and,} \tag{S39}$$

$$g_2 = G\left(\psi = \frac{1}{2}\right) = (h_{dd} + h_{uu} - 2h_{du}) = -8\left[G\left(\psi = \frac{1}{2}\right) - \frac{1}{2}(g_0 + g_1) + \ln(2)k_{\text{B}}T\right], \tag{S40}$$

from which h_{dd}, h_{uu}, and h_{du} follow. The results are summarized in Table S1 and S2, together with the degree of bistability λ, as calculated from Eq. 8 for $\psi = 0.5$.

Table S1: Fitting parameters and extracted interaction parameters *h*. The data set-crossing h-parameters are obtained by a global fitting approach. Accurate λ values are given in Table S2.

System	h_{dd} (meV)	h_{uu} (meV)	h_{du} (meV)	λ
TS_{tr} (Fig. 3d)	3.124 ± 0.023	4.036 ± 0.034	61.625 ± 0.142	> 1
H_{tr} high (Fig. 3e)	2.195 ± 0.013	6.560 ± 0.055	68.853 ± 0.070	> 1
H_{tr} low (Fig. 3e)	2.194 ± 0.025	2.391 ± 0.038	42.336 ± 0.512	< 1

We note that visually, the entropy-controlled data sets (Fig. 3d) have poorer agreement with the fits than the enthalpy-controlled ones (Fig. 3e). This is due to the fact that, for the time being, the model presented herein does not assume any temperature-dependence of the h-parameters, for which the only free fitting parameter for each individual data set is α. The h-parameters, on the other hand, must cover all data sets concurrently. The fitting of the enthalpy-controlled data sets is not subject to such constraints, which allows for the better match.

Supplementary Note 6: Doping Efficiency

Table S2: Extracted doping efficiencies α and degrees of bistability λ. λ is calculated following Eq. 8 with $\psi = 0.5$.

System	Temperature	α (eV V^{-1})	λ
TS_{tr} (Fig. 3d)	263 K	0.0078 ± 0.0001	1.281
	268 K	0.0161 ± 0.0001	1.257
	273 K	0.0179 ± 0.0001	1.234
	278 K	0.0138 ± 0.0001	1.212
	283 K	0.0132 ± 0.0001	1.191
	288 K	0.0177 ± 0.0001	1.170
	293 K	0.0211 ± 0.0001	1.150
	298 K	0.0264 ± 0.0002	1.131
	303 K	0.0320 ± 0.0003	1.112
H_{tr} high (Fig. 3e)	293 K	0.0223 ± 0.0002	1.277
H_{tr} low (Fig. 3e)	293 K	0.0506 ± 0.0005	0.793

As can already be inferred from the order of magnitude of the fundamental model equations, there is a scaling involved between theoretical and experimental findings. This is reflected by the α-parameters of Table S2. While these appear rather low at first glance, they can be readily understood by following reasoning:

Let the OECT gating be considered as the capacitive addition of charges. The charge carrier density n then changes as

$$n = n_0 + \frac{C^* \cdot V}{e} \tag{S41}$$

with the applied voltage V, where n_0 is the initial charge carrier concentration and C^* is the volumetric capacitance. Similarly, n is changed by changing the chemical potential μ, which can be expressed through the linear approximation of

$$n = n_0 + \left.\frac{dn}{d\mu}\right|_{\mu=\mu_0} \cdot (\mu_0 + \Delta\mu) + \mathcal{O}(\Delta\mu^n), \tag{S42}$$

where μ_0 is the initial chemical potential. The state occupation can be approximated by a Boltzmann distribution with

$$n = N_{\mathrm{eff}} \cdot \exp\left(\frac{E-\mu}{k_{\mathrm{B}}T}\right), \tag{S43}$$

where N_{eff} is the effective density of states in the valence band, E is the according energy, and μ is the Fermi level (i.e., chemical potential). Note that this expression refers to (electron) holes. With Eq. S42 it follows

$$n = \underbrace{N_{\mathrm{eff}} \cdot \exp\left(\frac{E-\mu_0}{k_{\mathrm{B}}T}\right)}_{n_0} - \frac{1}{k_{\mathrm{B}}T} \cdot \underbrace{N_{\mathrm{eff}} \cdot \exp\left(\frac{E-\mu_0}{k_{\mathrm{B}}T}\right)}_{n_0} \cdot (\mu_0 + \Delta\mu) \tag{S44}$$

$$= n_0 \left(1 - \frac{\mu_0 + \Delta\mu}{k_{\mathrm{B}}T}\right). \tag{S45}$$

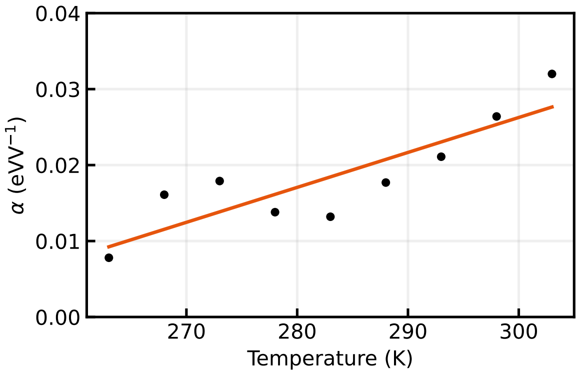

Fig. S17: Extracted and fitted doping efficiencies α. The doping efficiencies α from Table S2 are fitted with $\alpha(T) = k_\mathrm{B}Te^{-1} \cdot b + c$, according to Eq. S46 and S47 ($b = 8.55 \cdot 10^{-19}, c = -0.11$).

With Eq. S41, the change in chemical potential can then be linked to the applied voltage:

$$\mu_0 + \Delta\mu = -\frac{k_\mathrm{B}T}{e} \cdot \frac{C^*}{n_0} \cdot V \tag{S46}$$

$$= -\alpha \cdot V. \tag{S47}$$

Assuming a volumetric capacitance of $C^* = 40\,\mathrm{F\,cm^{-3}}$, a charge carrier concentration of $n_0 = 1{\cdot}10^{21}\,\mathrm{cm^{-3}}$, and $k_\mathrm{B}T = 25.7\,\mathrm{meV}$ ($T = 298\,\mathrm{K}$), the scaling factor results as $0.006\,\mathrm{eV\,V^{-1}}$, close to the estimated factors of Table S2. Note that this is only an approximate estimate to indicate the order of magnitude of the scaling factor. In fact, it is not unlikely that the actual volumetric capacitance is noticeably larger, as has been shown for the treatment of PEDOT:PSS with ionic liquids[6], which would result in values even closer to the ones found. The estimated relation also reflects the temperature-dependence of α we see in the data (Fig. S17), where the fitted factor of approximately 10^{-20} is sufficiently matching the ratio of C^* to n_0. Not surprisingly, the data from Table S2 finally also shows that the material composition has a notable influence on the doping efficiency (H_tr high vs. H_tr low). Not least, we infer a connection here to the capacitance described in Eq. S14.

Finally, it is worth pointing out that the above calculation is valid within the Boltzmann approximation, assuming an effective density of states model for the valence band. This applies to the on-state of the OECT, where the number of holes present in the valence band is lower than the effective density of states. However, this approximation does not hold in the subthreshold region, where the presence of intra-gap states (i.e., tail states) are causing an exponential rise in current. Here, a significantly higher doping efficiency must be assumed, which is reflected experimentally by the low subthreshold slope (e.g., Fig. 3h).

Supplementary Note 7: Effect of the Drain Voltage

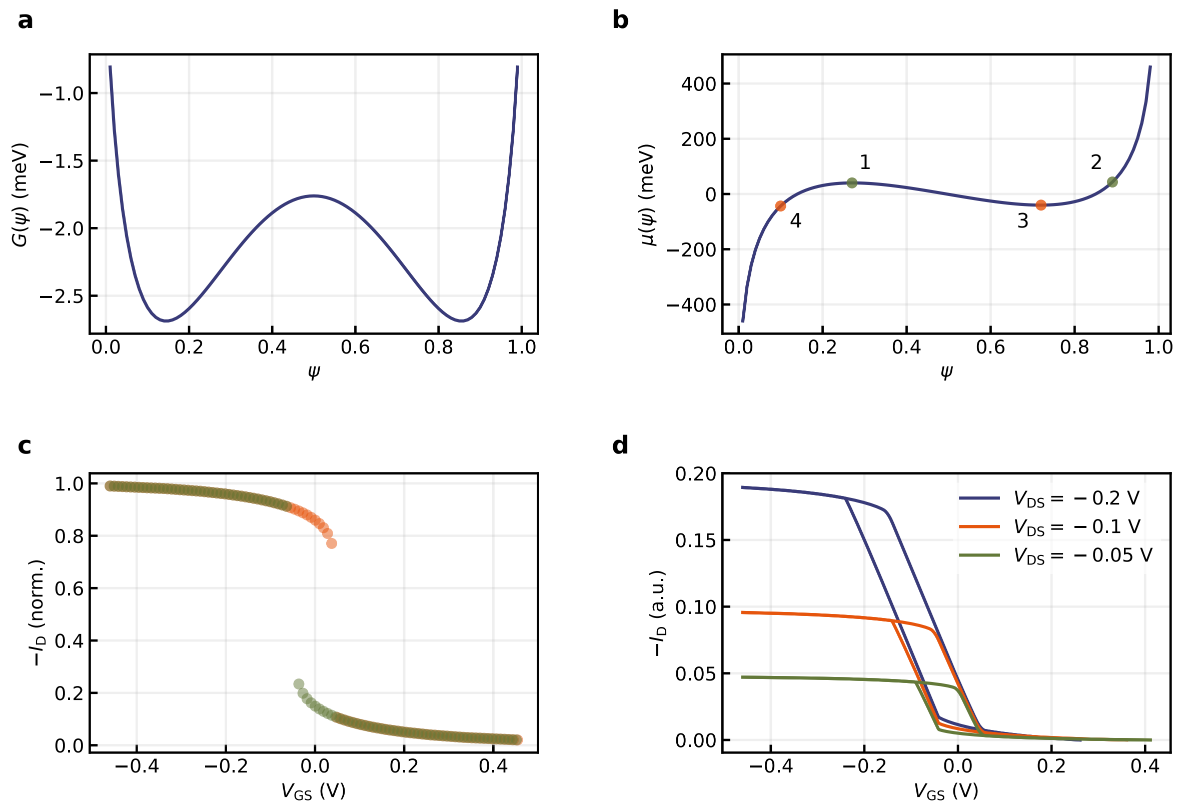

Fig. S18: Effect of the drain voltage. Given a bistability (**a**), the chemical potential is non-monotonic, with the four indicated points in (**b**) defining the transition points across the instable range of inverted slope. (**c**) During a transfer sweep, the physically stable points result in two branches for each sweep. Note that the orange path (dedoping) is partially covered by the green path (doping). (**d**) As the drain current is also a function of the drain voltage, the transition within each sweep deviates from a perpendicular leap.

The Gibbs free energy function of the bistable system results in a non-monotonic chemical potential profile, as derived above (Fig. S18a,b). Since the range of inverted slope is instable, there are four points to consider for understanding the shape of the experimental transfer curve. During doping ($\psi = 0 \to \psi = 1$), the profile of the chemical potential is followed until point 1 in Fig. S18b is reached. With V_{GS} being continuously raised, the next possible point on this curve is point 2, having the same chemical potential $\mu(\psi)$ at higher ψ. During dedoping ($\psi = 1 \to \psi = 0$), the same applies to points 3 and 4, respectively. This gives rise to two stable branches for each sweep, as shown in Fig. S18c. Note that the orange track (dedoping) is largely covered by the green track (doping). To understand the transition within each sweep, one needs to consider that the drain current I_{D} results from the integral of $\psi(\mu)$ from V_{GS} to $(V_{\mathrm{GS}} - V_{\mathrm{DS}})$, as discussed in Ref. 5. The slope of the transition is accordingly affected by V_{DS}, leading to a deviation from the perpendicular leap that one might expect from other bistable systems[7–9] (Fig. S18d).

Supplementary Note 8: Schmitt Trigger

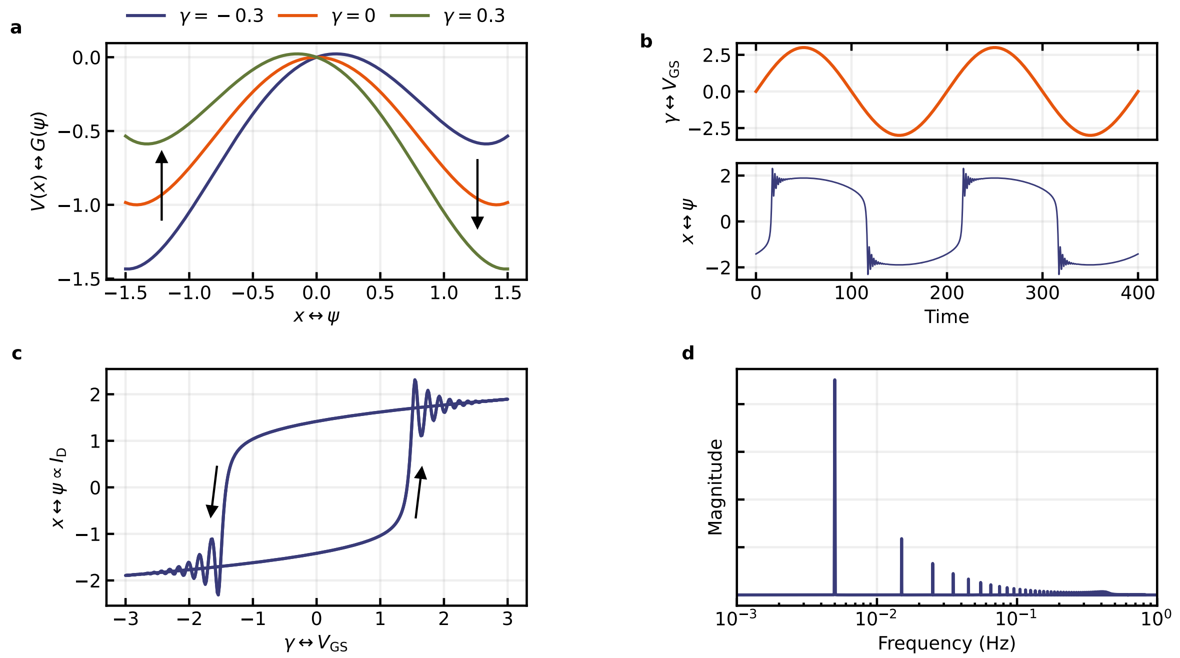

Fig. S19: Dynamic response of a bistable system. (**a**) $V(x)$ with $\alpha = 0.25, \beta = 0.5$ under an external bias γ. (**b**) Upper panel: Periodic external bias as defined in Eq. S51 with $A = 2, T = 200$. Lower panel: Time-dependent response of the system as calculated by solving the set of differential equations with $\epsilon = 0.5$. (**c**) Response of the system as a function of external bias. (**d**) Response of the system in frequency space, given by the Fourier transformation over a cycle of 100 periods.

We use the proxy system of Supplementary Note 2 to study the dynamics of the bistable OECT system. As before, we approach the Gibbs free energy function by the fourth order polynomial

$$V(x) = \alpha x^4 - \beta x^2 \quad \text{with} \quad \alpha = 0.25, \beta = 0.5. \tag{S48}$$

The dynamic response of this system is described by a set of two first-order differential equations,

$$\dot{x} = v \tag{S49}$$

$$\dot{v} = -\frac{\partial V(x)}{\partial x} - \epsilon v + \gamma, \tag{S50}$$

with the damping coefficient ϵ and the external driving force γ. The latter causes a deflection of the potential function, as shown in Fig. S19a. The external driving force can be considered as time dependent, for instance by means of a periodic signal

$$\gamma(t) = A \cdot \sin\left(\frac{2\pi t}{T}\right) \quad \text{with} \quad A = 2, T = 200, \tag{S51}$$

shown in Fig. S19b (upper panel). Together with a damping coefficient of $\epsilon = 0.5$, we solve the set of differential equations by numerical integration using the SciPy library[10]. Fig. S19b (lower panel) shows the time-dependent response of the system with corresponding damped

oscillations towards the upper and lower state. This reflects even more in Fig. S19c, showing the response as a function of external bias. In the frequency domain, the higher harmonics are clearly visible, as to be expected for a system of intrinsic bistability (Fig. S19d).

With this notion, we study the Fourier transformation of the experimental OECT data using a Fast-Fourier Transformation (FFT) algorithm. Therefore, the internal MATLAB function was used in the following form:

```
% RecordedWaveform consists of time values (column 1) and
   current values (column 3)
L=length(RecordedWaveform(1:end,1));
fs=abs(1./(RecordedWaveform(2,1)-RecordedWaveform(1,1))); %
   sample rate
f4 = (fs)*(0:(L/2))/L;
SpeIn4=fft(RecordedWaveform(1:end,3));
P2in = abs(SpeIn4/L);
P1in = P2in(1:L/2+1);
P1in(2:end-1) = 2*P1in(2:end-1);
```

Note that the FFT assumes the signal in time domain with infinite iterations, i.e., the first value of the recorded waveform is seen as the follow-up of the last and vice versa. Hence, the recorded waveform including beginning and ending should result in an integer value to avoid spurs in the spectrum. Furthermore, a higher number of cycles should be recorded for a higher precision. A longer recording duration leads to a higher accuracy at lower frequencies, while a higher sample rate increases the accuracy at higher frequencies. We considered the above mentioned points for the calculation of the spectrum shown in Fig. 4. Thus, we used a measurement consisting of 16 periods (653 seconds recorded) with a sample rate of 19.6 Hz as shown in Fig. S20a. The unfiltered spectrum is shown in Fig. S20b, showing higher harmonics, in line with our preliminary considerations of the proxy system.

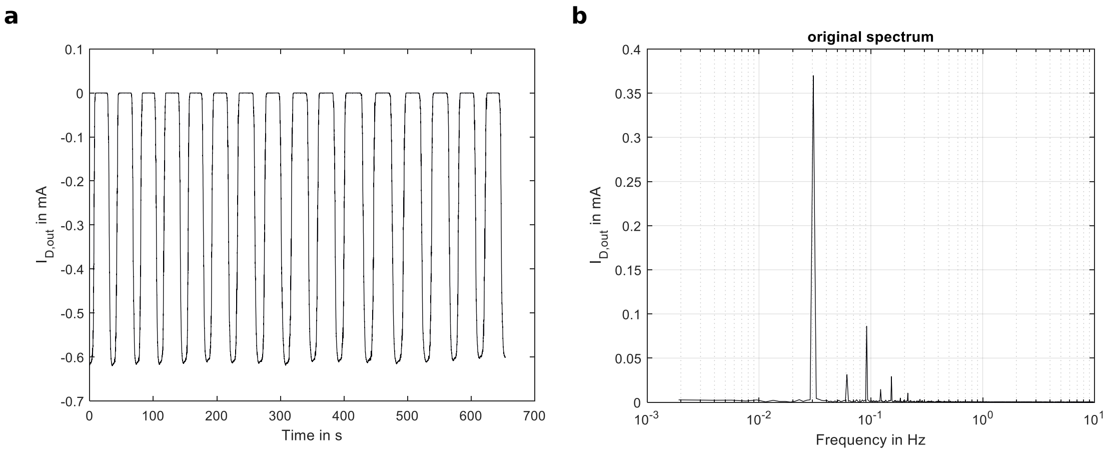

Fig. S20: OECT oscillation. (**a**) Recorded waveform of output current and (**b**) corresponding spectrum using an FFT algorithm in MATLAB.

For the filter design, we calculated several types of filters including higher order Butterworth

and Elliptic filters. However, for an easy hardware realization, a simple filter structure ought to be used, e.g., a first-order Butterworth filter. This filter can be realized utilizing only two passive components, e.g., one resistor and one capacitor. For the design, we again used an internal MATLAB function as follows:

```
fc4=6e-2; %corner frequency
[ym,xm] = max(P1in(2:end)); %skip dc
fc5 = f4(xm);
Wn4=2*fc4/fs;
[b4,a4] = butter(1,Wn4,'low'); %1st order Butterworth low-
   pass filter
nv4=filter(b4,a4,RecordedWaveform(1:end,3));
SpeOut4F=fft(nv4);
P2outF = abs(SpeOut4F/L);
P1outF = P2outF(1:L/2+1);
P1outF(2:end-1) = 2*P1outF(2:end-1);
[h_calc4, w_calc4] = freqz(b4,a4,L,fs); %filter's frequency
   response
```

This results in a filter function with a 3 dB-corner frequency of 60 mHz, a passband gain of 0 dB (passive filter), and a stop band attenuation of 20 dBdec^{-1} as shown in Fig. S21a. There, the filtered spectrum is also shown in frequency domain, while the filtered and unfiltered signals in time domain are shown in Fig. S21b.

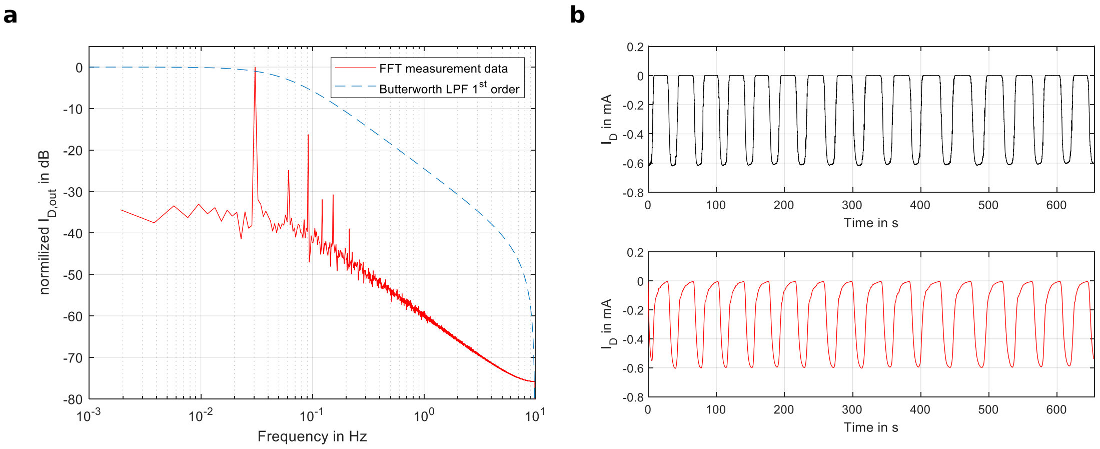

Fig. S21: First-order Butterworth filter. (**a**) Filtered spectrum in frequency domain using a first-order Butterworth filter (transfer function shown as dashed line) and recorded and filtered waveforms of the output current in time domain (**b**).

The obtained filter coefficient can be implemented, e.g., by a 24 kΩ resistor and a 1.1mF capacitor, both of which are commercially available.

Supplementary References

1. Bongartz, L. M. *Simulation Tool: Bistable Organic Electrochemical Transistors* `https://bit.ly/bistability`. 2024.
2. Bernards, D. A. & Malliaras, G. G. Steady-state and transient behavior of organic electrochemical transistors. *Advanced Functional Materials* **17,** 3538–3544 (2007).
3. Borgnakke, C. & Sonntag, R. E. *Fundamentals of Thermodynamics* (John Wiley & Sons, 2022).
4. Sze, S. M., Li, Y. & Ng, K. K. *Physics of Semiconductor Devices* (John Wiley & Sons, 2021).
5. Cucchi, M. *et al.* Thermodynamics of organic electrochemical transistors. *Nature Communications* **13,** 4514 (2022).
6. Wu, X. *et al.* Ionic-liquid doping enables high transconductance, fast response time, and high ion sensitivity in organic electrochemical transistors. *Advanced Materials* **31,** 1805544 (2019).
7. Dreyer, W. *et al.* The thermodynamic origin of hysteresis in insertion batteries. *Nature Materials* **9,** 448–453 (2010).
8. Koulakov, A. A., Raghavachari, S., Kepecs, A. & Lisman, J. E. Model for a robust neural integrator. *Nature Neuroscience* **5,** 775–782 (2002).
9. Vela, S. *et al.* The key role of vibrational entropy in the phase transitions of dithiazolyl-based bistable magnetic materials. *Nature Communications* **5,** 4411 (2014).
10. Virtanen, P. *et al.* SciPy 1.0: Fundamental Algorithms for Scientific Computing in Python. *Nature Methods* **17,** 261–272 (2020).
